# Book Reviews

**Published:** 1902-06

**Authors:** 


GENITO-URINARY DISEASES AND SYPHILIS. By Henry H. Morton, M.D.; Clinical Professor Genito-Urinary Diseases in the Long Island College Hospital. Surgeon to the Long Island College and Kings County Hospital and the Polhemus Memorial Clinic. Illustrated with Half-tones and Full-page Color-plates. The F. A. Davis Co., Publishers, Phila. 1902.

This work is designed to supply the general practitioner with concise information as to the present status of genito-urinary disease. Much progress has been made in this domain of medicine during the last ten years, and to classify and epitomize this progress is the function of the book. Considerable space is devoted to the treatment of enlarged prostate and to that of seminal vesiculitis, both of which conditions have received considerable attention within recent years. The book is up-to-date and will be welcomed as an addition to the literature on the subject.

---

THE PRACTICAL MEDICINE SERIES OF YEAR BOOKS. VOL. V. OBSTETRICS. Edited by Reuben Peterson, A.B., M.D., and Henry F. Lewis, A.B., M.D. Price, $1.25. The Year Book Publishers, 40 Dearborn St., Chicago.

This book is intended to present the views of the best writers who have considered the different branches of obstetrics in the last twelve months. The contents are made up of abstracts from articles which have appeared during the year. The field is very well covered. The volume presents our present knowledge of obstetrics in epitome and will doubtless prove of service.

